# Correction: Non-linear elastic properties of actin patches to partially rescue yeast endocytosis efficiency in the absence of the cross-linker Sac6

**DOI:** 10.1039/d2sm90083a

**Published:** 2022-06-24

**Authors:** Reda Belbahri, Alphée Michelot, Julien Heuvingh, Olivia du Roure

**Affiliations:** PMMH, CNRS, ESPCI Paris, Université PSL, Sorbonne Université, Université de Paris Paris France julien.heuvingh@espci.fr olivia.duroure@espci.fr; Aix Marseille Univ, CNRS, IBDM, Turing Centre for Living Systems Marseille France

## Abstract

Correction for ‘Non-linear elastic properties of actin patches to partially rescue yeast endocytosis efficiency in the absence of the cross-linker Sac6’ by Belbahri Reda *et al.*, *Soft Matter*, 2022, **18**, 1479–1488, https://doi.org/10.1039/D1SM01437D.

The authors wish to point out that in the author list of the published article, the author names were incorrectly presented, with family names incorrectly shown before given names. The corrected form of all of the author names is as shown above.

The Royal Society of Chemistry apologises for these errors and any consequent inconvenience to authors and readers.

## Supplementary Material

